# Clinical and epidemiologic characteristics associated with dengue fever in 2011–2016 in Bang Phae district, Ratchaburi province, Thailand

**DOI:** 10.1371/journal.pntd.0009513

**Published:** 2021-06-30

**Authors:** Jacqueline Kyungah Lim, Pornthep Chanthavanich, Kriengsak Limkittikul, Jung-Seok Lee, Chukiat Sirivichayakul, Kang Sung Lee, Sl-Ki Lim, In-Kyu Yoon, Weerawan Hattasingh

**Affiliations:** 1 International Vaccine Institute, Seoul, Republic of Korea; 2 Faculty of Tropical Medicine, Mahidol University, Thailand; 3 Coalition for Epidemic Preparedness Innovations (CEPI), Oslo, Norway; DoD - AFHSB, UNITED STATES

## Abstract

**Background:**

Dengue is a major public health problem in Thailand, but data are often focused on certain dengue-endemic areas. Methods: To better understand dengue epidemiology and clinical characteristics in Thailand, a fever surveillance study was conducted among patients aged 1–55 years, who presented with non-localized febrile illness at Bang Phae Community Hospital in Ratchaburi province, Thailand from October 2011 to September 2016.

**Results:**

Among 951 febrile episodes, 130 were dengue-confirmed. Individuals aged 10–14 years were mostly affected, followed by those 15–19 years-of-age, with about 15% of dengue-confirmed cases from adults 25 years and older. There were annual peaks of dengue occurrence between June-November. Most prevalent serotype in circulation was DENV-2 in 2012, DENV-3 in 2014, and DENV-4 & -3 in 2015. Among dengue cases, 65% were accurately detected using the dengue NS1 RDT. Detection rate was similar between secondary and primary dengue cases where 66% of secondary vs. 60% of primary dengue cases had positive results on the NS1 RDT. Among dengue cases, 66% were clinically diagnosed with suspected dengue or DHF, prior to lab confirmation. Dengue was positively associated with rash, headache, hematemesis and alterations to consciousness, when compared to non-dengue. Dengue patients were 10.6 times more likely to be hospitalized, compared to non-dengue cases. Among dengue cases, 95 were secondary and 35 were primary infections. There were 8 suspected DHF cases and all were identified to be secondary dengue. Secondary dengue cases were 3.5 times more likely to be hospitalized compared to primary dengue cases. Although the majority of our dengue-positive patients were secondary dengue cases, with few patients showing manifestations of DHF, our dengue cases were mostly mild disease. Even among children < 10 years-of-age, 61% had secondary infection and the rate of secondary infection increased with age.

**Conclusion:**

While the majority of dengue-confirmed cases were children, almost three-quarters of dengue-confirmed cases in this study were secondary dengue. Our study results consistent with previous data from the country confirm the hyperendemic transmission of DENV in Thailand, even in the non-epidemic years. With various interventions becoming available for dengue prevention and control, including dengue vaccines, decision-making on future implementation strategies should be based on such burden of disease data.

## Author summary

Dengue is a well-known public health problem in Thailand. To identify epidemiologic and clinical patterns of dengue in Thailand, we studied 951 febrile patients with fever, aged 1–55 years, who attended Bang Phae district hospital in Ratchaburi province, Thailand. Patients were tested with a rapid test for dengue, and further tests were carried out on paired blood samples taken 10–21 days apart. Overall, 14% of the febrile episodes identified between October 2011 and September 2016 were dengue-confirmed. Teenagers were mostly affected with only few adults. Dengue peaks occurred between June-November, but there was no large epidemic during the study period. DENV type 2 was the main serotype in circulation in 2012, DENV type 3 in 2014, and DENV types 4 and 3 in 2015. Dengue cases were 11 times more likely to be hospitalized than non-dengue cases. Dengue patients were more likely to present with rash, headache, hematemesis and alterations to consciousness, compared to non-dengue. Two thirds of the dengue cases were clinically diagnosed with suspected dengue or DHF, even without lab confirmation. Secondary dengue cases were about three times more common than primary cases. Even among children < 10 years-of-age, 61% of the patients were of secondary infection. Among dengue cases, 65% were accurately detected using the dengue RDT. This was not different between secondary and primary dengue cases. All 8 patients clinically diagnosed with DHF were secondary dengue cases. Although most of our dengue-positive patients were secondary dengue cases, we only observed few patients showing DHF. Thus, we concluded that our dengue cases were mostly of mild dengue illness. Our study results support the previous data confirming the high burden of dengue in Thailand and such information would help to make decisions on future implementation of various tools for dengue prevention and control.

## Introduction

Dengue fever (DF) is a mosquito-borne flavivirus infection caused by four related but antigenically distinct dengue viruses (DENVs, serotypes 1–4). The global burden of dengue has experienced a dramatic increase in recent years [1] and now DF with dengue hemorrhagic fever (DHF) are considered major causes of morbidity and mortality in tropical and sub-tropical countries [2,3]. With 390 million DENV infections estimated to occur annually worldwide, an estimated 500,000 severe dengue cases requiring hospitalization and approximately 20,000 deaths occur yearly [4–6].

As a major public health problem in Thailand, despite mosquito control efforts, DF/DHF has steadily increased in both incidence and range of distribution in the country. All four serotypes are in circulation and it has now spread to all provinces, districts, and sub-districts [7,8]. DHF is typically known to confined to children, but there is a shift in modal age with an increase in number of hospitalization due to DHF in older individuals [9,10]. With a well-established national dengue surveillance system, the incidence rates in Ratchaburi province are documented to be up to 698/100,000 person years.

There is a considerable amount of dengue burden data from Thailand based on epidemiological studies including those following dengue vaccine trials. However, often existing data are focused on hospitalized cases, despite that outpatient dengue accounts for the greatest burden of disease, both epidemiologically and economically. Thus, there continues to be a lack of data on dengue among non-hospitalized cases [11,12]. Also, despite the findings of clinical and epidemiological differences between adults and children, data on dengue among adults are relatively scarce compared to what is available for children [13,14].

In order to better understand epidemiologic and clinical patterns of symptomatic dengue in Bang Phae, a passive facility-based fever surveillance study was conducted in a catchment area among residents of Bang Phae district, Ratchaburi province, Thailand. The study served two objectives. First, dengue-confirmed cases were compared to non-dengue cases, to identify significant epidemiologic and clinical features associated with dengue-confirmation, including assessing patterns related to dengue rapid diagnostic test (RDT) results and clinical diagnosis. Secondly, among dengue-confirmed patients, differences between secondary and primary cases were assessed.

## Methods

### Ethics statement

The study protocol obtained ethical approvals from the Institutional Review Boards (IRBs) of the International Vaccine Institute (2011–007), the Ethics Committee of the Faculty of Tropical Medicine, Mahidol University (MUTM2011-031-06), and the Ethical Review Committee in Human Subjects (Ref.no.31/2554) of the Ministry of Public Health, Thailand.

All adult subjects provided written informed consent, and a parent or guardian of any child participant provided written informed consent on the child’s behalf with written assent from the child aged between 7 and 18 years.

### Site selection

In site selection, the factors such as reported incidence, cases, outbreaks in the literature, available seroprevalence studies, and adequate research infrastructure were considered [15–17]. Ratchaburi province ranks among the top ten provinces of Thailand for dengue incidence rates, ranging between 123.5–394.3/100,000 persons, with all 4 serotypes in circulation [18]. With the known high levels of DENV transmission and hyperendemicity, Bang Phae district, Ratchaburi province was selected in consultation with collaborators in Mahidol University and Ministry of Public Health (MoPH). The fever surveillance study was implemented in Bang Phae Community Hospital (BPCH).

### Study area and population

**Fig 1:**
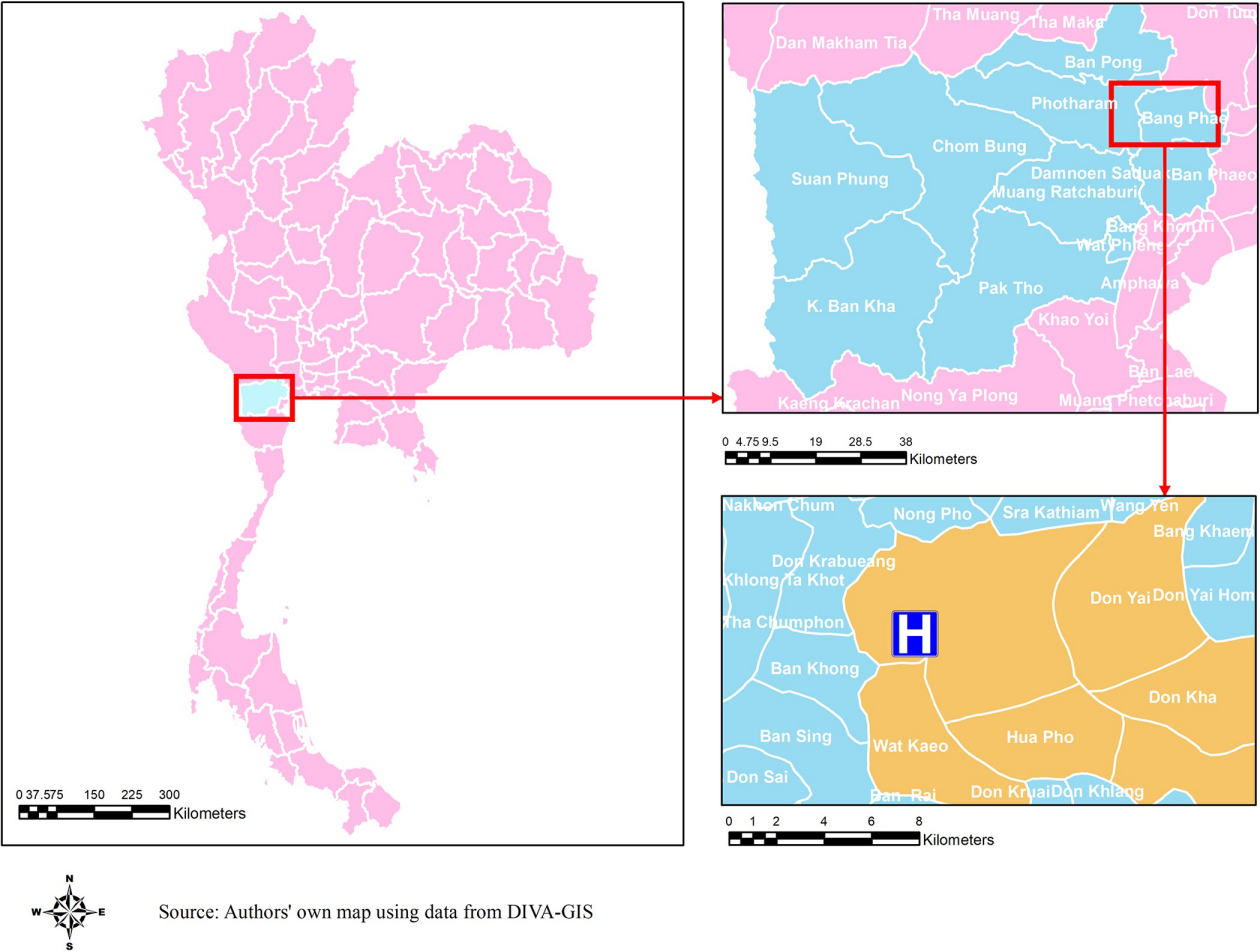
A map of the area of catchment population and study facility in Bang Phae district, Ratchaburi province, Thailand. A map of the study area in Bang Phae district, Ratchaburi province, Thailand. Base layer of the map can be found in https://www.diva-gis.org/gdata.

Ratchaburi is located approximately 85 km west to Bangkok. The population of the Ratchaburi province is 873,518 (2018)[19] with 31% of the population residing in the urban area (Fig 1). Bang Phae district of Ratchaburi province has 44,768 residents, approximately 11,000 households, over an area of 500 km^2^ (2017)[20]. Bang Phae district is composed of 65 villages within 7 sub-districts of Bang Phae, Wang Yen, Hua Pho, Wat Kaeo, Don Yai, Don Kha, and Pho Hak.

The primary healthcare provider in Bang Phae district is BPCH in Wang Yen sub-district. It is a 48-bed medium-sized secondary care facility which conducts up to 400 outpatient consultations per day (2011). There is a District Health Office and there are 9 sub-district health centers, with limited numbers of doctors on site, dispersed in the district.

To understand epidemiologic and clinical patterns of dengue fever in Bang Phae district, the passive facility-based fever surveillance was implemented in BPCH and study team staff enrolled outpatient and hospitalized patient with fever [15]. With unknown volume of potential eligible subjects presenting with fever at the hospital in the first year of the study period, every other eligible patient was recruited between October 2011 and September 2012. From October 2012 until the end of study period in September 2016, all eligible patients were recruited. Overall, the study continued for 5 years.

**Fig 2:**
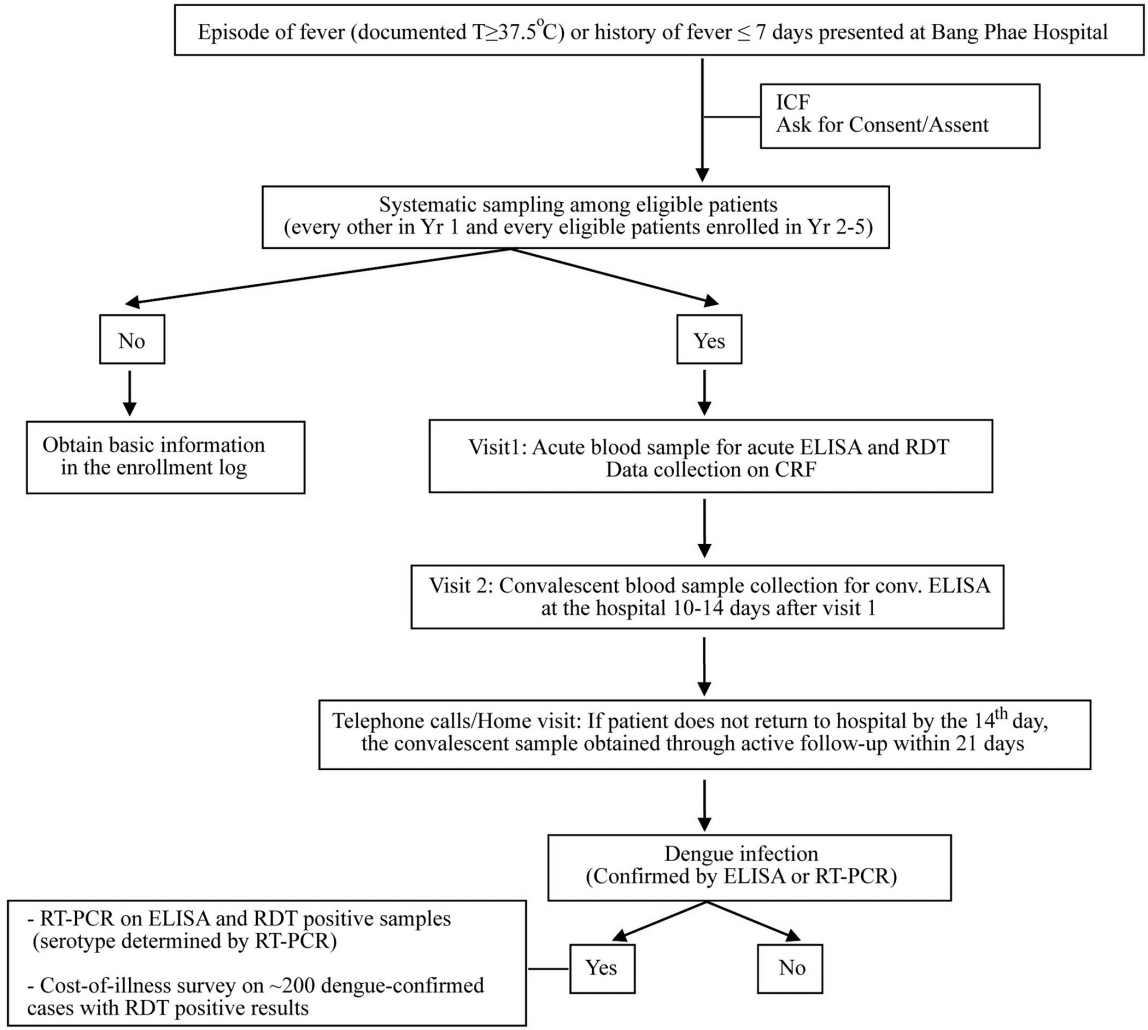
Passive fever surveillance algorithm at Bang Phae Community Hospital. Algorithm showing the design and flow of the passive fever surveillance.

When a febrile patient presented at the hospital, regular medical evaluation and case management of the patient were done by regular medical team at BPCH. And all febrile patients were referred to the study office within BPCH after the routine practice. After obtaining the informed consents, an acute blood sample of 5 ml was taken on the day of enrolment and tested with a commercial dengue RDTs (NS1 Antigen up to October 2013 and Dengue Duo with both NS1 and Immunoglobulin type (Ig) M cassettes from November 2013; Standard Diagnostics, Yongin-Si, Korea) on the visit 1. Also, the study staff collected basic demographic and clinical information, such as age, sex, address, medical history, symptoms and signs, treatment and laboratory results [21]. Patients were asked to return to the hospital for the convalescent sample collection between 10–14 days from visit 1. If the patients had not come to the hospital after the 14th day, the house visit was made within 21 days from visit 1. Confirmation of dengue infection was done using in-house enzyme-linked immunosorbent assay (ELISA) on paired samples collected on visits 1 and 2 [21,22]. In addition, samples were tested with reverse transcriptase-polymerase chain reaction (RT-PCR) for confirmation as well as to identify dengue serotypes. The study flow was shown in Fig 2.

### Study subjects

Eligible criteria for study enrollment:

1. Age 1–55 years;
2. Residents of the 7 sub-districts of Bang Phae district, Ratchaburi province;
3. Not participating in the ongoing dengue vaccine trials;
4. Signed informed consent from all adults >18, parental consent with participant assent for those aged between 7 and 18 years, and parental consent under age 7; and
5. Patients presenting with fever (Axillary temperature ≥ 37.5 celcius degrees (° C)) or history of fever for ≤ 7 days of duration without localizing signs (fever caused by a localized infection as well as fever with a known and confirmed etiology other than dengue).

### Laboratory testing algorithm

Acute samples were tested using a commercial RDT on the day of the first visit at BPCH. From BPCH, the acute and convalescent samples were centrifuged and sera were separated under sterile conditions, labeled, stored at -70°C freezer and then transported under freeze condition to TROPMED Dengue Diagnostic Center (TDC) at the Faculty of Tropical Medicine, Mahidol University. Sera samples were tested using in-house ELISA dengue IgM/IgG [23]. In addition, the samples of serological positive cases (i.e. RDT positive, positive IgM or significant increase in IgG titers) were tested with RT-PCR [24]. A small number of serological negative cases was also tested with RT-PCR. Also, acute samples were tested with in-house Japanese encephalitis (JE) IgM/IgG ELISA for check for possible cross-reactive IgM and IgG response.

Presence of positive IgM dengue specific antibody titer (>40 u/ml) by ELISA, virus detection (RT-PCR) in the acute serum specimen and at least 4-fold rise in DENV IgG titers from acute to convalescent phase sera were considered laboratory-confirmed dengue infection [25]. Primary dengue infection was defined by ELISA IgM-to-IgG ratio of ≥ 1.8, and secondary dengue infection by the ratio less than 1.8 [26].

### Statistical analysis

A descriptive summary of characteristics is presented by the dengue confirmation status (dengue-confirmed vs. non-dengue), as well as between secondary and primary cases among dengue-confirmed cases. Furthermore, in assessment of patterns of dengue, we also explored patterns of dengue RDT use, especially RDT results compared to lab-confirmation of dengue (by ELISA and/or PCR) and clinical suspicion of dengue, possibly leading to reduced antibiotics prescription. Clinical diagnosis prior to laboratory-confirmation, was grouped as suspected dengue, undifferentiated fever, and non-dengue. Categorical pair-wise comparisons were made across dengue status using *χ*^2^ or the Fisher’s exact tests with significance at *p*-value < 0.05. Comparison of continuous variables was performed using the student’s *t*-test and ANOVA.

A multivariable analysis was conducted to identify clinical indicators associated with dengue confirmation, using multivariable logistic regression models. Key parameters were identified in univariable associations with dengue confirmation status and between secondary and primary dengue cases. For a priori adjustments, the multivariable models were adjusted for the variables for age (a known confounder for dengue and its clinical presentation for primary infection vs. subsequent infections [27,28], further collapsed to 4-level categorical variable); gender (we had a priori reasons for believing that gender might be related to likelihood of exposure to the Aedes vectors and it might mediate some of the clinical presentations [29]) although in our data its inclusion or exclusion did not make difference to the odds ratios in the final model); timing of fever occurrence (whether the episode occurred during a known dengue season, often coinciding with a rainy period, may affect dengue diagnosis and physicians’ focus when making examinations for symptoms, etc. [30,31]); and, fever duration prior to visit (possibly affecting how symptoms are clinically presented at enrollment by the duration of illness and level of viremia for dengue confirmation [32]). Then, a multivariable backward stepwise logistic regression model was applied with a significance level 0.2 for entry and 0.1 for staying in the adjusted model. Adjusted for age, gender, timing of fever occurrence, and days of fever prior to visit, the independent variables included demographic and clinical variables such as treatment type (hospitalized patients vs. outpatients), clinical diagnosis, temperature measured at enrolment, and various signs and symptoms. From the multivariable backward stepwise logistic regression model, statistically significant variables were identified. These variables, with control variables and a priori adjustment, were entered in the final multivariable logistic regression to estimate association with dengue-confirmed against non-dengue cases. In addition, on a subset of dengue patients, a multivariable analysis to identify indicators associated with hospitalization among dengue patients, using logistic regression models. The significance level was set at *p*-value < 0.05. Associations were expressed in terms of odds ratios (ORs) with 95% confidence intervals (CIs). All analyses were performed using SAS version 9.4 (SAS Institute, Cary, North Carolina).

## Results

### General characteristics

**Fig 3:**
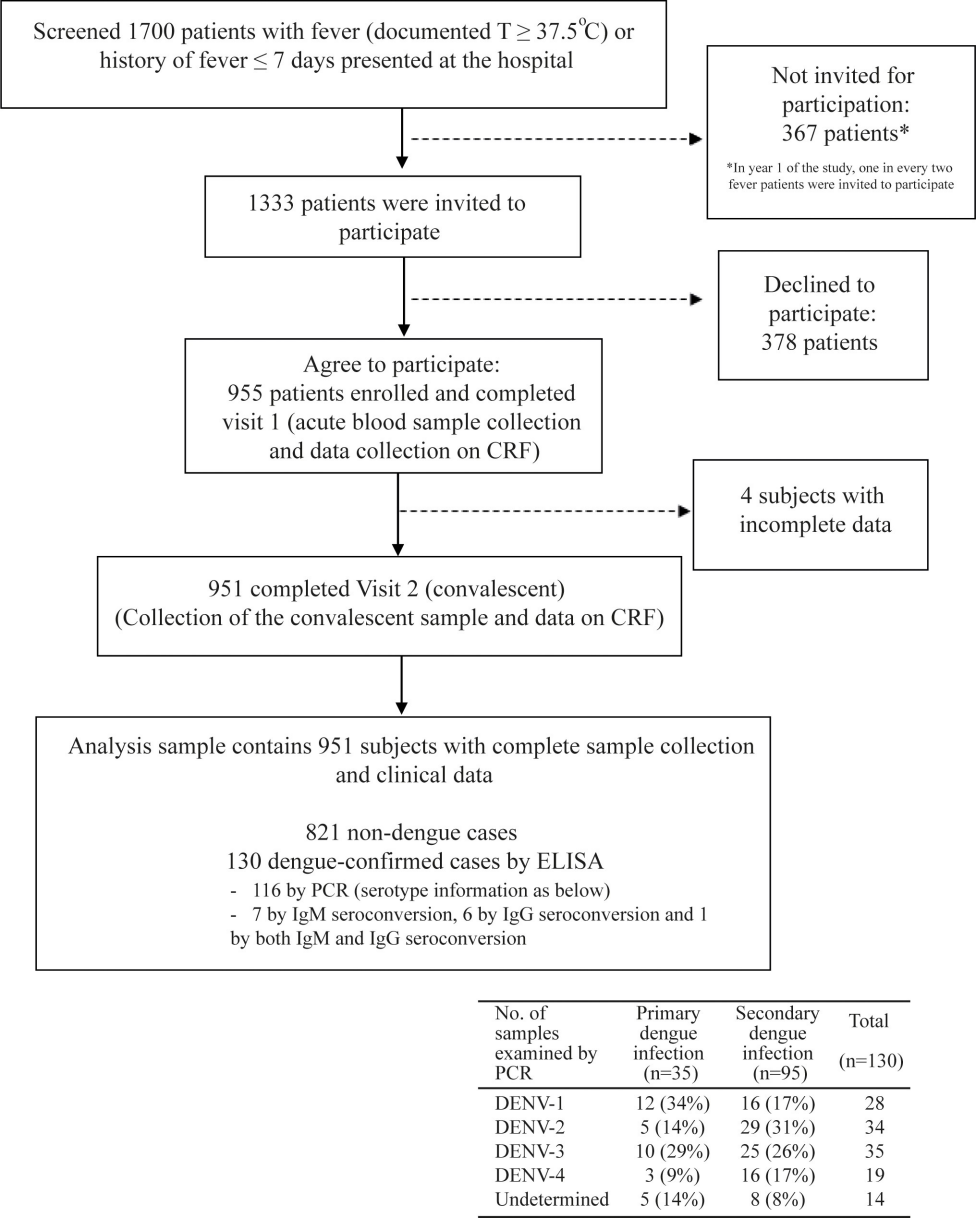
A chart of patient flow in the passive fever surveillance at the study facility. The chart describes patient flow in the passive fever surveillance from screening, enrollment to study participation, with determination of laboratory-based status of dengue infection, as well as how the analysis sample was reached.

Over the study period of 5 years, 1700 febrile patients presented at BPCH and 955 were enrolled (Fig 3). However, 4 subjects were excluded due to absence of visit 2. Therefore, 951 subjects who completed both visits 1 and 2 were included in the analysis sample.

**Fig 4:**
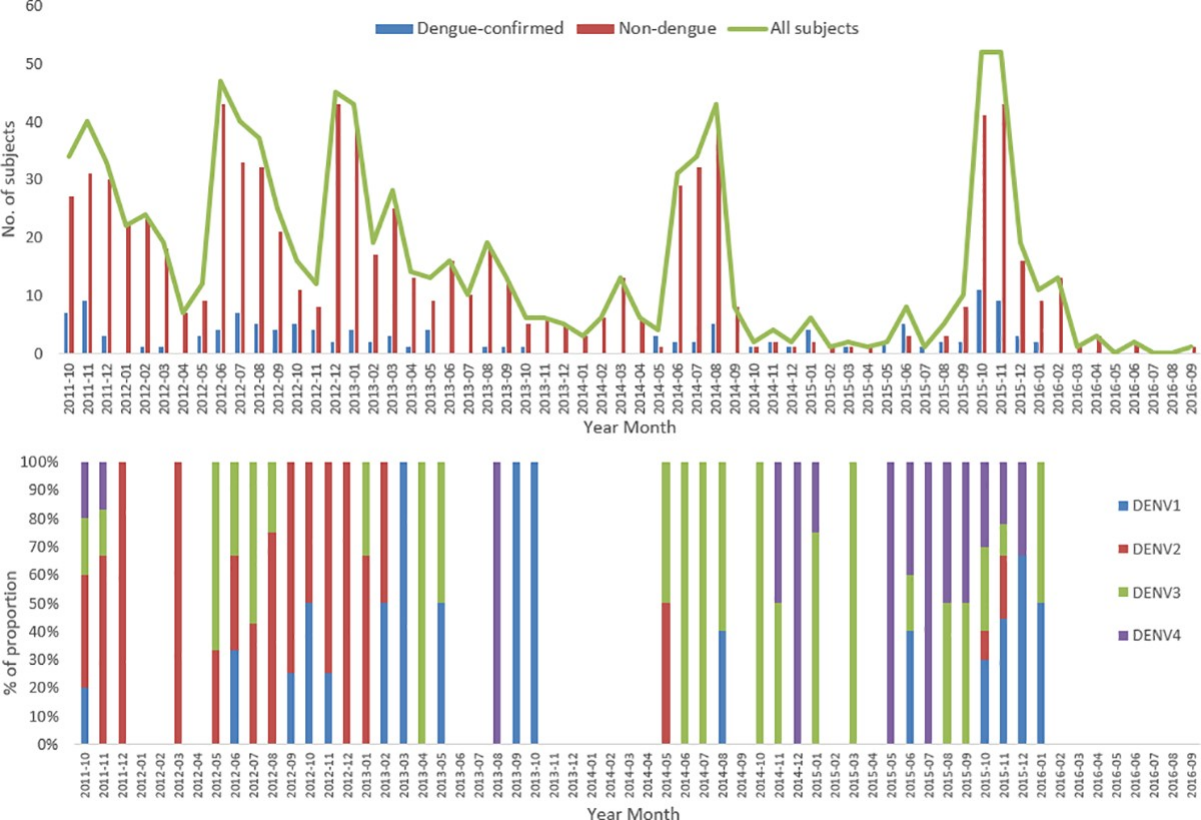
Monthly distribution of dengue and non-dengue cases among enrolled subjects during the study period. The figure shows monthly distribution of dengue-confirmed and non-dengue cases among the enrolled patients.

Among 951 febrile patients, 13.7% (n = 130) were found to be dengue-confirmed (Table 1). Of 130 dengue-confirmed patients, 95 were identified to be secondary and 35 to be primary dengue cases. There were annual peaks of dengue occurrence between June-November (Fig 4). In our surveillance, those that showed positive results on any of IgM/IgG ELISA and NS1 of the dengue RDT were tested with RT-PCR. Of 220 acute serum samples which underwent testing with RT-PCR, 116 were dengue-confirmed by PCR. The most prevalent serotype was DENV-3 (n = 35), followed by DENV-2 (N = 34). All four serotypes were identified during the study period, including DENV-1 (n = 28) and DENV-4 (n = 19). In 2012 and 2015, dengue peaks were larger than in 2013 and 2014. In 2012, it was mainly DENV-2 with some DENV-3 and, in 2015, it was mainly DENV-4 with some DENV-3 cases.

**Table 1:** Demographic and clinical characteristics of the dengue-confirmed and non-dengue patients as well as total enrolled study patients with non-localizing febrile illness from the health facility-based fever surveillance established in Bang Phae district, Ratchaburi province, Thailand in 2011–2016.

Characteristics (n; %)	Dengue-confirmed (n = 130)	Non-dengue (n = 821)	Total (n = 951)	p-value
**Age** (years)				
Mean (SD)	15.60 (9.82)	17.69 (15.22)	17.41 (14.62)	**< .001**
Range				**< .001**
1–4	7 (5.38)	138 (16.81)	145 (15.25)	
5–9	26 (20.0)	194 (23.63)	220 (23.13)	
10–14	40 (30.77)	153 (18.64)	193 (20.29)	
15–19	29 (22.31)	78 (9.50)	107 (11.25)	
20–24	9 (6.92)	39 (4.75)	48 (5.05)	
25–34	8 (6.15)	64 (7.80)	72 (7.57)	
35–44	9 (6.92)	68 (8.28)	77 (8.10)	
45–55	2 (1.54)	87 (10.60)	89 (9.36)	
**Female**	66 (50.77)	457 (55.66)	523 (54.99)	0.297
**Hospitalization**				**< .001**
Yes	51 (39.23)	47 (5.72)	98 (10.30)	
No	79 (60.77)	774 (94.28)	853 (89.70)	
Duration (mean days; SD)	4.10 (1.45)	3.54 (1.41)	3.82 (1.45)	**< .001**
Mean days of fever, prior to visit (SD)	3.35 (1.24)	3.38 (1.29)	3.37 (1.28)	**< .001**
**Duration of fever, prior to visit** (days)				
1–2	39 (30.0)	223 (27.16)	262 (27.55)	
3	32 (24.62)	251 (30.57)	283 (29.76)	
4–5	53 (40.77)	291 (35.44)	344 (36.17)	
6–7	6 (4.62)	56 (6.82)	62 (6.52)	
**Mean days of fever, entire illness** (SD)	6.48 (2.14)	5.70 (1.90)	5.81 (1.95)	**< .001**
**Mean temperature at presentation (°C)**	37.99 (1.07)	37.60 (0.89)	37.65 (0.93)	**< .001**
**Temperature at presentation**				
< 38.3	78 (60.0)	624 (76.0)	702 (73.8)	**< .001**
≥ 38.3	52 (40.0)	197 (24.0)	249 (26.2)	
**Prev. dengue infection**	6 (4.6)	45 (5.5)	51 (5.4)	0.684
JE vaccination**	114 (87.69)	646 (78.68)	760 (79.92)	**0.017**
Positive on either of IgM*** and/or NS1 of RDT	85 (65.38)	2 (0.24)	87 (9.15)	**< .001**
**Positive on the NS1 RDT**	84 (64.62)	2 (0.24)	86 (9.04)	**< .001**
**Primary/secondary dengue infection**				
Primary infection	35 (26.92)	-	35 (26.92)	-
Secondary infection	95 (73.08)	-	95 (73.08)	-

* An otic temperature ≥38.3°C, the 75th percentile of the body temperature for study patients measured at the time of enrolment, was used as the cutoff value to create a dichotomous variable indicating elevated body temperature.

**Dichotomous variables were created for Japanese Encephalitis vaccination history where those self-reported to have been vaccinated were grouped as one vs. the rest (i.e. those that did not remember or provided self-report that they have not had Japanese Encephalitis vaccination).

***599 subjects were tested with only NS1 RDT test (from start to October 2013); 352 subjects were tested with NS1 and IgM RDT test (from November 2013 until end of study)

The mean age for dengue-confirmed patients were significantly younger than the non-dengue patients (15.6 years vs. 17.7), even though most of our dengue-confirmed patients had secondary infection (Table 1). More than half of dengue-confirmed patients were teenagers between 10–19 years of age. And, about 15% of dengue-confirmed cases were adults 25 years and older. Of dengue-confirmed patients, 39% (51/79) were hospitalized, compared to only 6% (47/774) of non-dengue patients. Among 97 of 98 hospitalized patients with documented end date of hospitalization, mean duration of hospitalization was significantly longer for dengue-confirmed patients at 4.1 days, compared to non-dengue patients at 3.5 days.

Mean duration of fever prior to the first visit was about 3.4 days for both groups, but mean duration for entire illness was significantly longer for dengue-confirmed (6.5 days), compared to non-dengue, patients (5.7 days). There were 5% of enrolled patients with self-reported previous dengue infections. The percentage of subjects who self-reported having received JE vaccine was significantly higher in the dengue-confirmed patients (88%), compared to non-dengue patients (79%). When compared between those individuals who reported to have received JE vaccination as part of the expanded program on immunization (EPI) and those who reported to have received the JE vaccination outside of EPI, there was no difference in terms of proportion of dengue-positivity.

There were 59% of dengue-confirmed patients clinically diagnosed with suspected dengue fever and 6% with suspected DHF, whereas 91% of non-dengue patients were diagnosed with non-dengue (mostly upper respiratory illness (URI), viral syndrome, bronchitis) (Table 2). In terms of signs and symptoms, dengue-confirmed patients were significantly more likely to present with rash, fatigue, alteration of consciousness, headache, muscle pain, gum bleeding, and hematemesis, compared to non-dengue patients. Conversely, nasal congestion, rhinorrhea, sore throat, cough, and expectoration were found more commonly among non-dengue patients, compared to dengue-confirmed patients, with statistical significance.

**Table 2:** Clinical characteristics of the dengue-confirmed and non-dengue patients as well as total enrolled study patients with non-localizing febrile illness from the health facility-based fever surveillance established in Bang Phae district, Ratchaburi province, Thailand in 2011–2016.

Characteristics (n; %)				Dengue-confirmed (n=130)	Non-dengue (n=821)	Total (n=951)	p-value
**Clinical diagnosis**							
	Undifferentiated fever			6 (4.62)	56 (6.82)	62 (6.52)	**<.001**
	Suspected DF			77 (59.23)	13 (1.58)	90 (9.46)	
	Suspected DHF			8 (6.15)	2 (0.24)	10 (1.05)	
		Grade I/II		6/2	2/0	8/2	
	Non-dengue			39 (30.0)	750 (91.35)	789 (82.97)	
		URI		22 (56.41)	623 (83.07)	645 (81.75)	
		Viral syndrome		12 (30.77)	66 (8.80)	78 (9.89)	
		Bronchitis		2 (5.13)	23 (3.07)	25 (3.17)	
		Influenza		0	15 (2.0)	15 (1.90)	
		Others		3 (7.69)	23 (3.07)	26 (3.29)	
**Signs and symptoms**							
	Probable dengue						
		Nausea & vomiting		53 (40.77)	283 (34.47)	336 (35.33)	0.163
		Rash		18 (13.85)	25 (3.05)	43 (4.52)	**<.001**
	Ache and pain						
		Headache		117 (90.0)	597 (72.72)	714 (75.08)	**<.001**
		Retro-orbital pain		16 (12.31)	72 (8.77)	88 (9.25)	0.196
		Muscle pain		37 (28.46)	163 (19.85)	200 (21.03)	**0.025**
		Joint pain		6 (4.62)	31 (3.78)	37 (3.89)	0.646
	Positive tourniquet test			10 (7.7)	15 (1.8)	25 (2.6)	**<.001**
	Warning signs						
		Abdominal pain		15 (11.54)	64 (7.80)	79 (8.31)	0.151
		Oliguria		8 (6.15)	95 (11.57)	103 (10.83)	0.065
		Bleeding manifestations					
			Gum bleeding	2 (1.54)	1 (0.12)	3 (0.32)	**0.048**
			Hematemesis	14 (10.77)	21 (2.56)	35 (3.68)	**<.001**
		Fatigue/weakness		40 (30.77)	163 (19.85)	203 (21.35)	**0.005**
	Others						
		Alterations to consciousness		27 (20.77)	83 (10.11)	110 (11.57)	**<.001**
		Loss of appetite		50 (38.46)	317 (38.61)	367 (38.59)	0.974
	Respiratory						
		Breathing difficulty		2 (1.54)	22 (2.68)	24 (2.52)	0.441
		Nasal congestion		8 (6.15)	119 (14.49)	127 (13.35)	**0.009**
		Rhinorrhea		25 (19.23)	458 (55.79)	483 (50.79)	**<.001**
		Sore Throat		47 (36.15)	442 (53.84)	489 (51.42)	**<.001**
		Cough		49 (37.69)	651 (79.29)	700 (73.61)	**<.001**
		Sputum production		14 (10.77)	301 (36.66)	315 (33.12)	**<.001**
**Treatment prescribed**							**<.001**
	Antibiotics use			14 (10.77)	247 (30.09)	261 (27.44)	**<.001**
		Mean days of use (SD)		6.21 (5.22)	3.86 (2.52)	3.99 (2.77)	**<.001**
	Paracetamol use			130 (100.0)	821 (100.0)	951 (100.0)	-
		Mean days of use (SD)		5.28 (2.55)	4.64 (4.15)	4.73 (3.97)	**<.001**

Among dengue cases, 65% were accurately detected using the NS1 kit of the dengue RDT. This resulted in the sensitivity of 64.6% [95% Confidence Interval (CI): 55.8 to 72.8%] and the specificity of 99.8% (95% CI: 99.1 to 99.9%).

While non-dengue patients were almost 3 times more commonly prescribed with antibiotics than dengue-confirmed patients (30.1% of non-dengue patients vs. 10.8% of dengue-confirmed patients prescribed with antibiotics), mean duration of antibiotic use was significantly longer for dengue-confirmed patients than for non-dengue patients (6.2 days vs. 3.9 days, p-value < .001) (Table 2). Most of those who received antibiotics (257/261) had negative results on dengue RDT. Of 4 patients who received antibiotics with RDT positive results, although all clinical diagnosed with dengue, two were given longer than 7 days of antibiotics prescription compared to 25 of 253 individuals who were given longer than 7 days of antibiotics prescription with RDT negative results. Also, most of those prescribed with antibiotics (255/261) were clinically suspected with non-dengue.

Paracetamol was given to all enrolled subjects and mean duration of paracetamol use was longer among dengue-confirmed patients, compared to non-dengue patients. Ibuprofen was rarely prescribed, to about 1% of enrollees.

### Clinical characteristics of dengue-confirmed patients

From univariable analyses (S1 Table), variables that were found to be independently associated with dengue-confirmed patients when compared against non-dengue patients were: age, treatment type (hospitalized patients vs. outpatients), JE vaccination history, temperature at presentation, whether infection occurred during the known dengue peak season, rash, fatigue, alterations to consciousness, headache, nasal congestion, rhinorrhea, sore throat, cough, sputum production, muscle pain, and hematemesis. After checking for univariable relationships, the multivariable backward stepwise logistic regression models were run. Statistically significant variables selected were: age, treatment type, temperature at presentation, rash, headache, rhinorrhea, cough, alterations to consciousness, hematemesis, and oliguria.

To the clinical variables selected by the model, a priori adjustments (gender, dengue peak season, and fever duration prior to visit) were included in the final multivariable logistic regression model. Clinical diagnosis of suspected dengue (vs. undifferentiated fever or non-dengue) and positivity on RDT were initially considered potential confounders. However, they were too closely related to dengue-confirmation status as well as to signs and symptoms and were not entered in the final model. The final model, adjusted for a priori adjustments, showed that, there were 4.8 and 2.4 times greater odds of dengue-confirmed cases presenting with rash and headache, respectively, compared to non-dengue cases (Table 3). Also, dengue-confirmed cases were 4.8 and 2.2 times more likely to present with hematemesis and alterations to consciousness, respectively, compared to non-dengue cases. Dengue-confirmed patients were less likely to present rhinorrhea (OR = 0.39) and cough (OR = 0.27), compared to non-dengue patients.

**Table 3:** Multivariable logistic analysis showing significant indicators and their odds ratios of dengue confirmation in the health facility-based fever surveillance.

Characteristics	Multivariable analysis Dengue-confirmed vs. non-dengue		
	OR	95% CI	p-value
**Gender**			0.301
Male	Ref*	-	
Female	0.780	0.487–1.249	
**Age** (years)			**0.004**
1–9	Ref*	-	
10–14	2.513	1.357–4.653	
15–19	2.815	1.385–5.719	
20–34	1.437	0.639–3.231	
35–55	0.902	0.387–2.104	
**Hospitalization**			**< .001**
Yes	7.871	4.475–13.844	
No	Ref*	-	
Occurrence during the known peak season of dengue*			0.212
Outside the known season	Ref*	-	
June-November	1.376	0.833–2.274	
**Duration of fever, prior to visit** (days)			0.732
1–2	Ref*	-	
3	0.782	0.424–1.441	
4–7	0.904	0.525–1.558	
**Temperature at presentation** (Celsius)			**0.041**
Below 38.3	Ref*	-	
≥ 38.3	1.663	1.022–2.706	
**Presence of signs and symptoms (*ref*. *absence*)**			
Rash	4.796	2.152–10.686	**< .001**
Headache	2.431	1.190–4.968	**0.015**
Rhinorrhea	0.390	0.219–0.693	**0.001**
Cough	0.266	0.162–0.439	**< .001**
Alterations of consciousness	2.229	1.161–4.280	**0.016**
Hematemesis	4.764	1.903–11.923	**0.001**
Oliguria	0.398	0.156–1.013	0.053

*reference category

### Secondary and primary dengue cases

The majority of dengue-confirmed cases, 73% (n = 95), were identified to be secondary dengue infection. There was no statistically significant difference observed in age distribution between secondary and primary dengue patients (Table 4). Also, there was no significant difference in gender distribution between secondary and primary dengue patients.

**Table 4:** Demographic and clinical characteristics of patients with secondary vs. primary dengue infection among the dengue-confirmed cases from the health facility-based fever surveillance established in Bang Phae district, Ratchaburi province, Thailand in 2011–2016.

Characteristics (n; %)			Secondary dengue infection (n=95)	Primary dengue infection (n=35)	Dengue-confirmed (n=130)	p-value	Univariable analysis Secondary vs. primary dengue		
							OR	95% CI	p-value
Age (years)						0.216			0.252
	1-9		20 (21.1)	13 (37.1)	33 (25.4)		Ref*		
	10-14		31 (32.6)	9 (25.7)	40 (30.77)		2.24	0.81-6.20	
	15-19		20 (21.1)	9 (25.7)	29 (22.31)		1.44	0.51-4.14	
	20-34		14 (14.7)	3 (8.6)	17 (13.1)		3.03	0.73-12.67	
	35-55		10 (10.5)	1 (2.9)	11 (8.5)		6.50	0.74-56.99	
Female			47 (49.5)	19 (54.3)	66 (50.77)	0.626	0.83	0.38-1.79	0.627
Hospitalization									
	No		51 (53.7)	28 (80.0)	79 (60.77)	**0.001**	Ref*		
	Yes		44 (46.3)	7 (20.0)	51 (39.23)		**3.45**	**1.37-8.67**	**0.008**
	Days of stay (mean; SD)		4.17 (1.46)	3.71 (1.38)	4.10 (1.45)	**<.001**			
Duration of fever, prior to visit (days)									
	Mean (SD)		3.21 (1.15)	3.74 (1.40)	3.35 (1.24)	**<.001**			0.266
	3 (ref. 1-2 days)						0.92	0.29-2.89	
	4-7						0.50	0.20-1.30	
Duration of fever, entire illness (days)									
	Mean (SD)		6.44 (2.18)	6.59 (2.05)	6.48 (2.14)	**<.001**			
Temperature at presentation (°C)						**0.044**			0.105
	< 38.3		52 (54.7)	26 (74.3)	78 (60.0)		Ref*		
	≥ 38.3		43 (45.3)	9 (25.7)	52 (40.0)		1.91	0.87-4.19	
High body temp at enrollment *(ref*. *lower temp)*							2.39	1.01-5.64	0.047
JE vaccination			82 (86.3)	32 (91.4)	114 (87.69)	0.431	0.59	0.16-2.21	0.435
Positive on the NS1 of RDT			63 (66.3)	21 (60.0)	84 (64.6)	0.504	1.31	0.59-2.92	0.505
Clinical diagnosis									
	Suspected dengue		66 (69.5)	19 (54.3)	85 (65.4)	0.107	1.92	0.87-4.25	0.109
		Suspected DF/DHF	58/8	19/0	77/8		-	-	-
	Other than dengue		29 (30.5)	16 (45.7)	45 (34.6)		Ref*		
Signs and symptoms (ref. absence)									
	Rash		11 (11.6)	7 (20.0)	18 (13.85)	0.218	0.52	0.19-1.48	0.223
	Fatigue/weakness		34 (35.8)	6 (17.1)	40 (30.77)	**0.041**	**2.69**	**1.02-7.14**	**0.046**
	Alterations of consciousness		22 (23.2)	5 (14.3)	27 (20.77)	0.269	1.81	0.63-5.22	0.273
	Headache		87 (91.6)	30 (85.7)	117 (90.0)	0.323	1.81	0.55-5.97	0.328
	Retro-orbital pain		13 (13.7)	3 (8.6)	16 (12.31)	0.431	1.69	0.45-6.33	0.435
	Rhinorrhea		17 (17.9)	8 (22.9)	25 (19.23)	0.524	0.74	0.29-1.90	0.525
	Sore Throat		35 (36.8)	12 (34.3)	47 (36.15)	0.788	1.12	0.50-2.52	0.788
	Cough		36 (37.9)	13 (37.1)	49 (37.69)	0.938	1.03	0.46-2.30	0.938
	Nausea & vomiting		45 (47.4)	8 (22.9)	53 (40.77)	**0.012**	**3.04**	**1.25-7.37**	**0.014**
	Abdominal pain		9 (9.5)	6 (17.1)	15 (11.54)	0.113	0.51	0.17-1.54	0.231
	Loss of appetite		42 (44.2)	8 (22.9)	50 (38.46)	**0.026**	**2.68**	**1.10-6.49**	**0.030**
	Muscle pain		33 (34.7)	4 (11.4)	37 (28.46)	**0.009**	**4.12**	**1.34-12.68**	**0.014**
	Joint pain		6 (6.3)	0	6 (4.62)	0.146	-		
	Hematemesis		11 (11.6)	3 (8.6)	14 (10.77)	0.624	1.40	0.37-5.33	0.625

*reference category

Among hospitalized patients, 86% (44/51) were secondary dengue patients. Patients with secondary dengue infections were 3.5 (95% CI: 1.37–8.67) times more likely to be hospitalized, compared to those with primary dengue infection. Duration of hospitalization was measured among 49 patients and the mean duration was significantly longer for those with secondary (4.17 days) vs. primary dengue infection (3.71 days).

Secondary dengue infections were more likely to be diagnosed as suspected dengue, but the difference in proportions was not statistically significant, when compared against primary dengue infections. Furthermore, all those clinically diagnosed with DHF among dengue-confirmed cases (n = 8) had secondary dengue and all of these secondary cases were hospitalized.

Among secondary dengue cases, 66.32% (95% CI: 55.89% - 75.69%) had positive results on the NS1 kit of RDT, compared to 60.0% (95% CI: 42.11% - 76.13%) in primary dengue cases. However, the difference was not statistically significant (p = 0.504). In terms of symptomatic presentation, secondary dengue cases were significantly more likely to present fatigue (O.R = 2.7 times), nausea/vomiting (O.R = 3.0 times), loss of appetite (O.R = 2.7 times), and muscle pain (O.R = 4.1 times), compared to primary dengue cases. There were no hemorrhagic signs that were found significantly more among secondary dengue cases, compared to primary dengue cases.

## Discussion

Dengue is well studied in many of Southeast Asian countries, including Thailand. There have been extensive studies conducted to assess epidemiologic patterns and burden of dengue disease in the country. Our study results showed consistent data with previous studies. We found 14% (n = 130) of 951 enrolled febrile patients to be dengue-confirmed. Similarly, Sabchareon et al. reported 2.7% to 10.2% of the febrile episodes to be dengue-confirmed among primary school students in Muang district of Ratchaburi province between 2006 and 2009 [33].

While there are dengue epidemics that occur every 2–4 years, during our study period, there was no large epidemic of dengue in Bang Phae district. Nonetheless, Also, as previously documented, there were annual peaks of dengue occurrence between June-October/November [34,35]. There were bigger peaks in 2012 and 2015 and smaller peaks were observed in 2013–14. Based on serotyping information from RT-PCR, it seems that a shift from DENV-2 to DENV-3 occurred causing a steep increase in cases, observed as the peak in 2012. DENV-4 reappeared in November 2014 and made up most of the peak in 2015 with DENV-3. While the most commonly found serotypes were DENV-3 and DENV-2, there were all 4 serotypes circulating in Bang Phae district during the study years confirming that it is an area with dengue hyperendemicity. As shown in our study, previous studies reported all DENVs in continuous circulation in Thailand [36,37].

### Patterns of dengue-confirmed cases in terms of age

In terms of age, more than 50% of the febrile patients enrolled at BPCH were under 15 years of age. More than 50% of those dengue-confirmed were 10–19 years of aged. A study conducted in Photharam Hospital in Ratchaburi in 2005–2015 also showed similar age patterns where 40% of dengue patients were found to be between 10–18 years of age with those between 10–14 years mostly affected [34]. Similarly, a study calculated dengue incidence in northeastern Thailand in 2006–2016 using the national surveillance system and also found the highest proportion of dengue cases to be in 5–14 years age group [38].

With the majority of these dengue-confirmed patients as secondary dengue cases, our data showed that there was no statistically significant difference in age distribution between secondary and primary dengue cases. Among patients with secondary dengue infections, the youngest individual was 2 years old (PCR confirmed with DENV-1) and reached up to 45 years-of-age. Such patterns with respect to age are consistent to what have been reported from other studies conducted in Thailand [34,36,38].

### Use of dengue RDT, clinical diagnosis, and antibiotics

In our studied population, we observed a high index of clinical suspicion of dengue with 66% of dengue-confirmed patients clinically diagnosed with dengue infection. The large majority of non-dengue cases had respiratory infections, including URI, viral syndrome, bronchitis. While these may seem atypical manifestations of dengue, our surveillance only excluded febrile patients if they have an obvious localized infection.

While clinical diagnosis of suspect dengue was made prior to lab confirmation, the RDT test results were available during visit 1. With the NS1 kit of the dengue RDT demonstrating 65% sensitivity and specificity of 99%, clinical judgement would have been made in the presence of knowledge of the dengue RDT results. When we consider either IgM or NS1 results on the RDT, of 87 patients with RDT positive results, 92% (n = 80) were diagnosed with suspected dengue or DHF. In terms of specificity of clinical diagnosis, 98% (n = 844) of 864 patients with RDT negative results were diagnosed with non-dengue. When clinical diagnosis of dengue was compared against lab-confirmation of dengue, sensitivity was quite high at 65.38% (95% C.I = 56.54–73.51) and specificity was even higher at 98.17% (95% C.I = 97.00–98.97). Even if aided by RDT results, in our studied population, we observed clinical diagnosis to perform well in detection of dengue cases.

Furthermore, dengue RDTs have been reported to be more sensitive for primary than secondary infections [39]. However, RDT performance (referring to the NS1 results only to be consistent throughout the study), compared against dengue RDT positivity determined by paired ELISA and RT-PCR results as the gold standards in our study, was similar in secondary (66.3%) and primary (60.0%) dengue cases with no significant difference. Consequently, secondary and primary dengue cases were similarly diagnosed with suspected dengue. Also, RDT performance could be influenced by days since onset of fever of the patients in different groups. However, our data did not show any difference in mean number of days since onset of fever between those with positive vs. negative results on RDT.

In the current study, we referred to only the NS1 results for assessment of the test performance of the dengue RDT. However, to compare with the clinical diagnosis, we referred to positivity either based on IgM and/or NS1 kit of the RDT and there may be concerns of cross-reactivity between flaviviruses reported in antibody assays and tests for dengue NS1 antigen [40,41]. However, quoted accuracy of the SD Dengue Duo NS1 antigen and IgG/IgM combo test is high: with the sensitivity and specificity of 92.8 and 98.4, respectively, for the NS1 side; 99.4 and 93.0, respectively, for the IgM/IgG side [42]. Also, previous studies support that commercial kits based on dengue NS1 antigen detection perform well in clinical samples from areas with multiple flaviviruses in circulation [41,43]. Furthermore, in the current study, the RDTs were used for screening, rather than for confirmation of dengue infection.

In terms of treatment, all patients were given paracetamol and the period of prescription was not significantly different between dengue and non-dengue patients. For antibiotics, prescription was more common among non-dengue (30%), than dengue-confirmed cases (11%). The mean duration of antibiotic use was almost twice longer for dengue-confirmed than non-dengue patients. Of 14 dengue-confirmed cases, the reasons for doctors giving antibiotics were negative RDT results and suspected bacterial infection including URI (n = 8).

### Symptomatic patterns of dengue-confirmed patients

In terms of symptomatic presentation, the final model reported that dengue-confirmed cases were significantly associated with increased odds of presenting with rash, headache, hematemesis and alterations to consciousness, while dengue-confirmed patients were significantly associated with decreased odds of presenting with rhinorrhea and cough, compared to non-dengue patients. In addition to being part of the 2009 WHO case definition of probable dengue, our results were consistent to what was commonly reported symptoms observed among patients with dengue fever in Thailand [18,44–46]. Dengue may not be commonly associated with neurological manifestations and alterations to consciousness was found to be positively associated with dengue in our model. In our study, alteration of consciousness was found only brief and non-progressive drowsiness, without severe neurological manifestations (stupor, coma, etc.).

In addition to these signs and symptoms, our model reported that dengue-confirmed cases were more likely to present with higher body temperature ≥ 38.3°C at enrollment than non-dengue cases. This was found statistically significant even after adjusting for fever duration days prior to visit and supported by other previous studies [18,45].

### Severe cases and secondary dengue

Hospitalization due to dengue illness is a common indicator of morbidity [47] and number of hospitalizations has been reported to be increasing in patients with dengue fever [48]. In our data where the mean age of hospitalized patients (n = 51, 16.24 years) was not significantly different from that of outpatients (n = 79, 15.19 years), the model showed that the dengue-confirmed patients were 8 times more likely to be hospitalized, compared to non-dengue patients. While this could have been an indicator of a severe outcome due to dengue infection, our dengue-confirmed patients reported a mean of 4.1 days of hospitalization compared to 3.5 days for non-dengue patients. Despite the statistically significant difference, the mean duration of hospital stay was longer for dengue-confirmed patients only by about a half day.

Other indicators of possible severe outcomes of dengue could be the warning signs [46]. The 1997 dengue classification system was originally divided into DF/DHF/Dengue shock syndrome (DSS), and it was revised in 2009 to be divided into: dengue fever, dengue fever with warning signs, and severe dengue, to cover for those cases that cannot be classified into DF/DHF/DSS [46,49]. In terms of discharge diagnosis, of 130 lab-confirmed dengue cases in our data, 77 were diagnosed with DF and 8 were diagnosed with DHF. All 8 DHF patients had secondary dengue infection. Among those DHF patients, 6 patients were classified as DHF grade I and 2 with grade II. There was no case of death.

Given the hyperendemicity of dengue in Thailand, it is expected that most of the cases are secondary dengue infections. Our data showed a significantly higher rate of admission, 3.5 times more likely, and a significantly longer mean duration of hospitalization among secondary cases compared to primary dengue cases in the studied population. However, given that our data did not have many cases with warning signs or cases diagnosed with DHF, most of patients in the studied population showed to have mild disease of dengue.

### Study limitations

We recognize variability of dengue epidemiology over time and by region. While the surveillance continued for 5 years and BPCH is the main hospital serving the studied population, due to resource constraints, subject recruitment was only at one facility. In addition, depending on the transmission volume of dengue or other co-circulating pathogens, there may have been cases of co-infection. There is documented co-circulation of multiple arboviruses, such as chikungunya virus (CHIKV), and Zika virus (ZIKV), observed in Thailand [50]. However, lab testing algorithm in this study did not cover other arboviruses than DENV and JEV. In a previous study conducted in Southern Thailand, among 163 DENV positive cases, 6 cases of co-infection with CHIKV and 1 case with ZIKV [50]. While it may be important to test for other key arboviruses to accurately measure the burden of dengue, we anticipate a minimal rate of possible co-infection cases.

An important potential source of bias is under-ascertainment due to the community residents with relevant symptoms not seeking care. In its passive design, our study may miss those eligible patients seeking for care at other healthcare providers than the facility under surveillance. Also, we did not recruit more mild patients who may seek care at private clinics. So, there may have been limitations in capturing of the wide spectrum of clinical manifestations of dengue by only involving a district-level hospital. We recognize that there may be limited generalizability due to these limitations.

## Conclusion

Our findings confirm the hyperendemicity of dengue with all 4 serotypes in circulation in Bang Phae, Ratchaburi province, Thailand, even in the times without large reported epidemics. While mild illness was observed in the majority of our dengue patients, we found more secondary cases than primary dengue cases even in teenagers. Given the importance of the burden data in making decisions on interventions, our results could contribute to facilitate decision-making on implementation of interventions, especially given that more options are becoming available for preventive and control measures, including vaccines.

## Supporting information

S1 Table Univariable analysis showing significant indicators and unadjusted odds ratios of dengue confirmation, comparing dengue-confirmed (n = 130) to non-dengue patients (n = 821) among the subject of the health facility-based fever surveillance. (DOCX)

S1 Strobe Checklist Clinical and epidemiologic characteristics associated with dengue fever in 2011–2016 in Bang Phae district, Ratchaburi province, Thailand. (DOCX)
