# Supplementary material for: Clinical and epidemiologic characteristics associated with dengue fever in 2011–2016 in Bang Phae district, Ratchaburi province, Thailand
Source: PLoS Negl Trop Dis. 2021 Jun 30;15(6):e0009513. doi: 10.1371/journal.pntd.0009513 (PMC8244866; doi:10.1371/journal.pntd.0009513)
Supplement: S1 Table — (DOCX) [file pntd.0009513.s001.docx]

S1 Table. Univariable analysis showing significant indicators and unadjusted odds ratios of dengue confirmation, comparing dengue-confirmed (n=130) to non-dengue patients (n=821) among the subject of the health facility-based fever surveillance

| **Characteristics**  **(n; %)** | **Total (N)** | **Dengue- confirmed** | **Non-dengue** | **Univariable analysis**  **Dengue-confirmed vs. Non-dengue** | | | |
| --- | --- | --- | --- | --- | --- | --- | --- |
|  |  |  |  | **OR** | **95% CI** | **p-value** | |
| **Gender** |  |  |  |  |  | 0.298 | |
| Male | 428 | 64 (15.0) | 364 (85.1) | Ref | - |  | |
| Female | 523 | 66 (12.6) | 457 (87.4) | 0.82 | 0.57-1.19 |  | |
| **Age** (years)** |  |  |  |  |  | **<.001** | |
| 1-9 | 365 | 33 (9.0) | 332 (91.0) | Ref | - |  | |
| 10-14 | 193 | 40 (20.7) | 153 (79.3) | 2.63 | 1.60-4.33 |  | |
| 15-19 | 107 | 29 (27.1) | 78 (72.9) | 3.74 | 2.14-6.53 |  | |
| 20-34 | 120 | 17 (14.2) | 103 (85.8) | 1.66 | 0.89-3.10 |  | |
| 35-55 | 166 | 11 (6.6) | 155 (93.4) | 0.71 | 0.35-1.45 |  | |
| **Hospitalization**** |  |  |  |  |  | **<.001** | |
| Yes | 98 | 51 (52.0) | 47 (48.0) | Ref | - |  | |
| No | 853 | 79 (9.3) | 774 (90.7) | 10.63 | 6.72-16.82 |  | |
| **JE vaccination**^A^* |  |  |  |  |  | **0.019** | |
| No/unknown | 191 | 16 (8.4) | 175 (91.6) | Ref | - |  | |
| Yes | 760 | 114 (15.0) | 646 (85.0) | 1.93 | 1.11-3.34 |  | |
| **Duration of fever, prior to visit**  (days) |  |  |  |  |  | 0.385 | |
| 1-2 | 262 | 39 (14.9) | 223 (85.1) | Ref | - |  | |
| 3 | 283 | 32 (11.3) | 251 (88.7) | 0.73 | 0.44-1.20 |  | |
| 4-7 | 406 | 59 (14.5) | 347 (85.5) | 0.97 | 0.63-1.51 |  | |
| **Temperature at presentation****  (Celsius) |  |  |  |  |  | **<.001** | |
| Below 38.3 | 702 | 78 (11.1) | 624 (88.9) | Ref | - |  | |
| ≥ 38.3 | 249 | 52 (20.9) | 197 (79.1) | 2.11 | 1.44-3.11 |  | |
| **Occurrence during the known peak season of dengue** (June-November)* |  |  |  |  |  | **0.027** | |
| Outside the peak season | 377 | 40 (10.6) | 337 (89.4) | Ref | - |  | |
| Peak season | 574 | 90 (15.7) | 484 (84.3) | 1.57 | 1.05-2.33 |  | |
| **Signs and symptoms** |  |  |  |  |  |  | |
| Probable dengue |  |  |  |  |  |  | |
| Nausea & vomiting | 336 | 53 (15.8) | 283 (84.2) | 1.31 | 0.90-1.91 | 0.164 | |
| Rash** | 43 | 18 (41.9) | 25 (58.1) | 5.12 | 2.71-9.68 | **<.001** | |
| Ache and pain |  |  |  |  |  |  | |
| Headache** | 714 | 117 (16.4) | 597 (83.6) | 3.38 | 1.87-6.11 | **<.001** | |
| Retro-orbital pain | 88 | 16 (18.2) | 72 (81.8) | 1.46 | 0.82-2.60 | 0.198 | |
| Muscle pain* | 200 | 37 (18.5) | 163 (81.5) | 1.61 | 1.06-2.44 | | **0.026** |
| Joint pain | 37 | 6 (16.2) | 31 (83.8) | 1.23 | 0.50-3.02 | | 0.646 |
| Positive tourniquet test | 25 | 10 (40.0) | 15 (60.0) | 4.10 | 1.79-9.37 | **0.001** | |
| Warning signs |  |  |  |  |  |  | |
| Abdominal pain | 79 | 15 (19.0) | 64 (81.0) | 1.54 | 0.85-2.80 | 0.154 | |
| Oliguria | 103 | 8 (7.8) | 95 (92.2) | 0.50 | 0.24-1.06 | 0.070 | |
| Bleeding manifestations |  |  |  |  |  |  | |
| Hematemesis** | 35 | 14 (40.0) | 21 (60.0) | 4.60 | 2.28-9.29 | **<.001** | |
| Fatigue/weakness* | 203 | 40 (19.7) | 163 (80.3) | 1.79 | 1.19-2.70 | **0.005** | |
| Others |  |  |  |  |  |  | |
| Alterations to consciousness** | 110 | 27 (24.6) | 83 (75.5) | 2.33 | 1.44-3.77 | **<.001** | |
| Loss of appetite | 367 | 50 (13.6) | 317 (86.4) | 0.99 | 0.68-1.45 | 0.974 | |
| Respiratory |  |  |  |  |  |  | |
| Breathing difficulty | 24 | 2 (8.3) | 22 (91.7) | 0.57 | 0.13-2.44 | 0.447 | |
| Nasal congestion* | 127 | 8 (6.3) | 119 (93.7) | 0.39 | 0.18-0.81 | **0.012** | |
| Rhinorrhea** | 483 | 25 (5.2) | 458 (94.8) | 0.19 | 0.12-0.30 | **<.001** | |
| Sore Throat** | 489 | 47 (9.6) | 442 (90.4) | 0.49 | 0.33-0.71 | **<.001** | |
| Cough** | 700 | 49 (7.0) | 651 (93.0) | 0.16 | 0.11-0.23 | **<.001** | |
| Sputum production** | 315 | 14 (4.4) | 301 (95.6) | 0.21 | 0.12-0.37 | | **<.001** |

Statistical significance of the frequencies: *p-value<0.05 **p-value<.001

^A^based on self-report
